# The Prevention of Laceration of the Perineum

**Published:** 1858-12

**Authors:** 


*The Prevention of Laceration of the Perineum.*—The *Brit. and For. Med. Chir. Rev.* gives the following views upon the prevention of laceration of the perineum, by Matte, (Vierteljahrsschrift, f. p. Heilk, 1858.)

"It is especially necessary that the head pass the vulva in a favorable direction. This can only happen when it passes with the necessary degree of flexion, whilst the occiput passes under the pubic arch, the face has not yet quitted the pelvic outlet; first, when the upper part of the neck comes under the pubic arch, can the extension of the head (or the separation of the chin from the breast) begin. If the distension of the perineum begins too early, the head must pass the vulva with unfavorable diameters—namely, with the great oblique, or great or straight diameters. Such a passage easily causes laceration.—Hence it is the business of the physician to prevent a premature distension by the head. This he effects by placing two fingers between the labia, or in some cases between the pubic arch and occiput, so as to bring the head downwards and outwards, at the same time laying the other hand on the hinder part of the perineum, upon which the face is lying, and push this upwards. This manœuvre is to be executed during the pains, which will thus protrude the head forwards in the requisite arc. A very simple means of expediting the birth of the head, consists in compressing firmly, the distended perineum with the whole hand. This resembles the squeezing out of the kernel from the cherry. On the passage of the shoulders, care must also be taken lest the two shoulders pass together.—*Medical & Surgical Reporter.*

